# Effects of cotton straw added to total mixed pelleted feed on the composition of rumen microbiota in breeding ewes

**DOI:** 10.3389/fmicb.2025.1604710

**Published:** 2025-08-08

**Authors:** Zhijun Zhang, Duanji Sang, Junyu Zhang, Lingling Su, Amat Guzalnur, Liangzhong Hou, Tongjun Guo

**Affiliations:** ^1^Feed Research Institute of Xinjiang Uygur Autonomous Region Academy of Animal Sciences, Ürümqi, China; ^2^Key Laboratory of Herbivorous Livestock Feed Biotechnology, Ürümqi, China

**Keywords:** cotton straw, breeding ewes, rumen microbiota, dietary inclusion levels, microbial diversity

## Abstract

This study aimed to investigate the effects of adding increasing amounts of cotton straw to the feed of breeding ewes on the bacterial and fungal microbiota of the rumen. A single-factor, completely randomized design was adopted, in which 120 ewes of small-tailed Han sheep were randomly divided into six groups, 20 ewes per group. Control group 1 (CK1) was fed a diet without cotton straw, while control group 2 (CK2) contained cottonseed meal in place of cotton straw. The experimental groups were supplemented with 20% (M20 group), 30% (M30 group), 40% (M40 group), or 50% (M50 group) cotton straw. The trial included a 30-day pre-trial period and a 270-day formal trial period. The results showed that, among the rumen bacterial microbiota, there was no significant difference in the number of operational taxonomic units (OTUs) between the groups. The ACE and Chao biodiversity indices of the CK2 group and M20-M50 groups were significantly lower than those of the CK1 group. Principle coordinate analysis (PCoA) of bacterial diversity revealed clear separation between the CK2 group and the M20-M50 groups compared with the CK1 group. With respect to rumen fungal microbiota, the M20 and M50 groups had the highest number of unique OTUs. There were no significant differences in abundance and diversity indices, and PCoA showed no obvious clustering differences among the groups. In summary, supplementing the basal diet with different proportions of cotton straw reduced the diversity of rumen bacteria and altered the composition of rumen bacterial and fungal communities. Adding more than 40% cotton straw significantly increased the abundance of Proteobacteria, which could have adverse effects on sheep growth performance.

## 1 Introduction

Cotton, as one of the world’s leading economic crops, not only holds a pivotal position in the textile industry but also produces huge amounts of waste products such as cotton straw, which is a potential agricultural resource. According to the latest statistical data in China, the cotton planting area in 2023 reached 2.788 million hectares (The National Bureau of Statistics, 2023). Based on a yield of 2,398–4,197 kg per hectare, the annual production of cotton straw in China is approximately 6.69 to 11.7 million tons, making it a major crop source of roughage after rice straw, corn straw, and wheat straw. However, due to its high lignin and free gossypol content, 400–600 ppm of free gossypol has caused toxicity in young ruminants, as well as issues such as lack of processing technology, unclear addition proportions, and safety concerns, cotton straw faces significant limitations as a feed additive. These factors not only affect the fermentation efficiency of rumen microorganisms but may also have adverse effects on the health of ruminants. Therefore, how to effectively utilize this resource and improve its nutritional value and safety in feed has become an important topic in the development of sustainable animal husbandry and agriculture.

Studies have shown that pelleting cotton straw after crushing can prevent selective feeding by sheep and decrease waste, but the heat generated during the processes of grinding and pelleting can degrade free gossypol (Liangzhong et al., 2020). Determining the appropriate addition ratio of cotton straw in pelleted ruminant diets and enhancing its utilization efficiency are of great significance for alleviating forage shortages, especially in maj1or cotton-producing regions. When cotton straw made up 50% of the dry matter in the diet for fattening sheep, the daily weight gain was similar to that of the control group without cotton straw (Lingling et al., 2020). Further research revealed that as the cotton straw content in the diet for fattening sheep increased, serum levels of aspartate aminotransferase, alanine aminotransferase, creatine kinase, and lactate dehydrogenase gradually increased, while urea nitrogen levels showed a downward trend (Zhang et al., 2020). In terms of rumen fermentation, a 40% addition of cotton straw significantly reduced the levels of ammonia nitrogen, isobutyric acid, isovaleric acid, and valeric acid in rumen fluid (Amat et al., 2020). Cotton straw additions of 40 and 50% significantly increased the dry matter and crude protein content in the muscles of fattening sheep, and as the cotton straw content increased, the levels of myristic acid and palmitic acid in lamb meat also increased (Duanji et al., 2021). Studies have shown that adding cotton straw to pelleted diets had no significant impact on reproductive performance of breeding ewes (Junyu et al., 2020a). However, when the cotton straw addition approached 50%, the serum urea nitrogen content in lactating ewes significantly decreased on day 7 of lactation; the 40 and 50% groups were significantly decreased at day 60 of lactation (Junyu et al., 2020b). Rumen microorganisms play a crucial role in the production efficiency and health of ruminants, and the rumen microbial community and its structure are closely related to growth performance and rumen fermentation parameters, making it essential to determine the optimal amount of cotton straw roughage to add to pelleted diets.

Current research has provided evidence on the production performance of breeding ewes, but there has been limited reporting on the effects of dietary cotton straw on their rumen microbial community. Therefore, this study aimed to investigate the impact of different ratios of cotton straw added to pelleted diets on the rumen microbial structure of breeding ewes, providing a theoretical basis for the scientific application of cotton straw in the production practices of breeding ewes.

## 2 Materials and methods

### 2.1 Characteristics of ewes and experimental design

A total of 120 healthy, small-tailed Han ewe lambs with similar ages (~7 months) and an average body weight of 42.3 kg, with a deviation of 6.11 kg, were obtained from Jinhuitong Industrial Development Co., Ltd. (Yuepuhu County, China). After deworming, the ewes were divided into six groups using a single-factor completely randomized design, with 20 ewes in each group. Control group 1 (CK1) was fed a basic diet without cotton straw and control group 2 (CK2) was fed the basic diet containing cottonseed meal but no cotton straw. Based on the CK2 diet, the experimental groups M20, M30, M40, and M50 were supplemented with 20, 30, 40, and 50% cotton straw, respectively. The trial period included mating and continued for 60 days after lambing, totaling 300 days, with a 30-day pre-trial period and a 270-day formal trial period.

### 2.2 Experimental sheep diet

Free gossypol can affect the reproductive performance of ewes. In preliminary research, the effects of cotton straw addition on the conception rate, lambing rate, and birth weight of lambs in replacement ewes were investigated. This study, based on previous findings, investigates the impact of different levels of cotton straw addition on the rumen microbiota of breeding ewes. With reference to China’s “Feeding Standards for Meat Sheep” (NY/T 816–2004), diets were formulated following the principle of equal energy and nitrogen to meet the nutritional requirements of ewes pregnant with twin lambs. The diet compositions and nutritional levels for the early gestation period (0–3 months) and late gestation period (4–5 months) are shown in Tables 1, 2. Coarse feeds (roughage) such as corn stalks, cotton straw, wheat straw, and alfalfa were passed through a 2 cm sieve and mixed uniformly with other ingredients. After adjusting the moisture content, the mixture was processed into complete pelleted feed with a diameter of 8 mm using a ring die pellet mill.

**Table 1:** Composition and nutrient levels of diets for ewes in early gestation (%, dry matter basis).

Feedstock	CK1	CK2	M20	M30	M40	M50
Diet composition						
Corn	21.50	22.60	21.40	20.20	19.30	18.30
Wheat bran	3.00	2.20	3.40	4.90	5.90	6.70
Cottonseed meal	0.00	5.60	5.60	5.30	5.20	5.10
Soybean meal	5.90	0.00	0.00	0.00	0.00	0.00
Premix^a^	2.00	2.00	2.00	2.00	2.00	2.00
NaHCO_3_	0.20	0.20	0.20	0.20	0.20	0.20
NaCl	0.40	0.40	0.40	0.40	0.40	0.40
Cotton straw	0.00	0.00	20.00	30.00	40.00	50.00
Corn stalks	40.00	40.00	20.00	10.00	0.00	0.00
Wheat straw	17.80	16.00	17.90	18.50	19.40	13.50
Alfalfa hay	9.20	11.00	9.10	8.50	7.60	3.80
Nutrient levels^b^						
Metabolizable energy (ME), MJ/kg	6.69	6.69	6.69	6.69	6.69	6.69
Crude protein	8.55	8.15	8.53	8.82	9.08	9.47
Ether extract	1.96	1.94	1.24	1.31	1.49	1.46
Neutral detergent fiber	44.08	44.69	41.47	37.08	40.98	37.60
Acid detergent fiber	28.60	26.67	27.35	24.57	29.08	26.18
Ca	0.71	0.52	1.26	0.83	1.10	1.02
P	0.17	0.21	0.18	0.21	0.23	0.24
Free gossypol (mg/kg)	36	59	101.3	103.6	130.5	148.9
lignin	6.92	9.95	7.34	14.58	13.84	13.73

a The premix provided the following nutrients per kg: VitA 200,000 IU, VitD3 30,000 IU, VitE 250 IU, nicotinic acid 500 mg, pantothenic acid 150 mg, biotin 10 mg, Cu 100 mg, Fe 1,200 mg, Mn1000 mg, Zn 1,000 mg, I17.5 mg, Se 7.5 mg, Co 7.5 mg.

b ME was a calculated value while the others were measured values.

**Table 2:** Composition and nutrient levels of diets of ewes in late gestation (%, dry matter basis).

Feedstock	CK1	CK2	M20	M30	M40	M50
Diet composition						
Corn	35.60	38.20	36.80	36.50	36.20	35.50
Wheat bran	2.20	1.60	2.50	2.00	1.80	2.80
Cottonseed meal	0.00	10.30	10.00	10.00	10.00	10.10
Soybean meal	10.00	0.00	0.00	0.00	0.00	0.00
Premix^a^	2.50	2.50	2.50	2.50	2.50	2.50
NaHCO_3_	0.30	0.30	0.30	0.30	0.30	0.30
NaCl	0.40	0.40	0.40	0.40	0.40	0.40
Cotton straw	0.00	0.00	20.00	30.00	40.00	48.40
Corn stalks	40.00	40.00	20.00	10.00	0.00	0.00
Wheat straw	3.00	2.00	3.20	3.70	4.40	0.00
Alfalfa hay	6.00	4.70	4.30	4.60	4.40	0.00
Nutrient levels^b^						
Metabolizable energy/(MJ/kg)	8.00	8.00	8.00	8.00	8.00	8.00
Crude protein	8.62	8.77	8.07	8.36	8.58	8.82
Ether extract	2.61	2.83	3.23	3.26	3.18	3.81
Neutral detergent fiber	38.54	44.64	41.12	41.12	40.37	40.92
Acid detergent fiber	18.90	20.66	20.33	21.30	20.34	20.60
Ca	0.60	0.70	0.88	0.91	0.90	0.95
P	0.25	0.28	0.26	0.24	0.26	0.28

a The premix provided the following nutrients per kg: VitA 200,000 IU, VitD3 30,000 IU, VitE 250 IU, nicotinic acid 500 mg, pantothenic acid 150 mg, biotin 10 mg, Cu 100 mg, Fe 1,200 mg, Mn1000 mg, Zn 1,000 mg, I17.5 mg, Se 7.5 mg, Co 7.5 mg.

b ME was a calculated value while the others were measured values.

### 2.3 Feeding and management of experimental sheep

The test ewes were fed twice a day at 09:00 am and 19:30 PM, ate and drank freely, and retained 15% of the leftovers every day. In summer, the enclosure was disinfected and sprayed to kill mosquitoes and flies, and the test ewes were in the sports field during the spraying. Artificial estrus was induced for natural mating on day 30 of the preliminary test period. The lambs were weaned at 60 days of age.

### 2.4 Sample collection and 16S rDNA sequencing

At day 300 of the test period, three test sheep from each group were slaughtered in a fasted state before morning feeding. After slaughter, the rumen digesta were collected and filtered through four layers of gauze. The filtrate was snap frozen in liquid nitrogen and stored at-80°C for later extraction of rumen microbial DNA. Nucleic acid extraction was completed by using the TGuide S96 genomic DNA extraction kit (manufacturer: Tiangen Biochemical Technology (Beijing) Co., LTD., model: DP812). The concentration of the extracted nucleic acid was measured with a Synergy HTX microplate reader (Gene Co., Ltd). The amplified PCR products were tested by electrophoresis on a 1.8% agarose gel (Beijing BMW Fuxin Technology Co.). The target fragment of ruminal fluid DNA amplification was the V3 + V4 region of the rDNA using PCR primers: 338\u00B0F: 5′ -ACTCCTACGGGAGGCAGCA-3′ and 806 R:5′ -GGACTACHVGGGTWTCTAAT-3′. For constructing libraries the amplicons were sequenced using an Illumina NovaSeq6000. The resulting sequences were assigned to an operational taxonomic unit (OTU) using USEARCH (version 10.0) software. The classification annotation of the OTU was performed using the Naive Bayes classifier in the QIME2 software, and the classification was based on the Silva database (version 138.1) with a confidence threshold of 70%. Alpha diversity determination was performed using the QIME2 software. In addition, one-way analysis of variance (ANOVA) was used to compare the relative abundance and diversity differences of microorganisms. Linear discriminant analysis (LDA) and linear discriminant analysis effect size (LEfSe) were used to evaluate the differential abundance of microorganisms.

### 2.5 Statistical analysis of the data

The data were analyzed by ANOVA using SPSS ver25.0, and multiple comparisons with Duncan’s post-hoc test were conducted to determine the significance of differences. A *p* < 0.05 was considered significant and *p* < 0.01 was deemed extremely significant.

## 3 Results and analysis

### 3.1 Analysis of rumen bacteria

#### 3.1.1 Composition and abundance of rumen bacteria in sheep fed with different percentages of cotton straw

**Figure 1:**
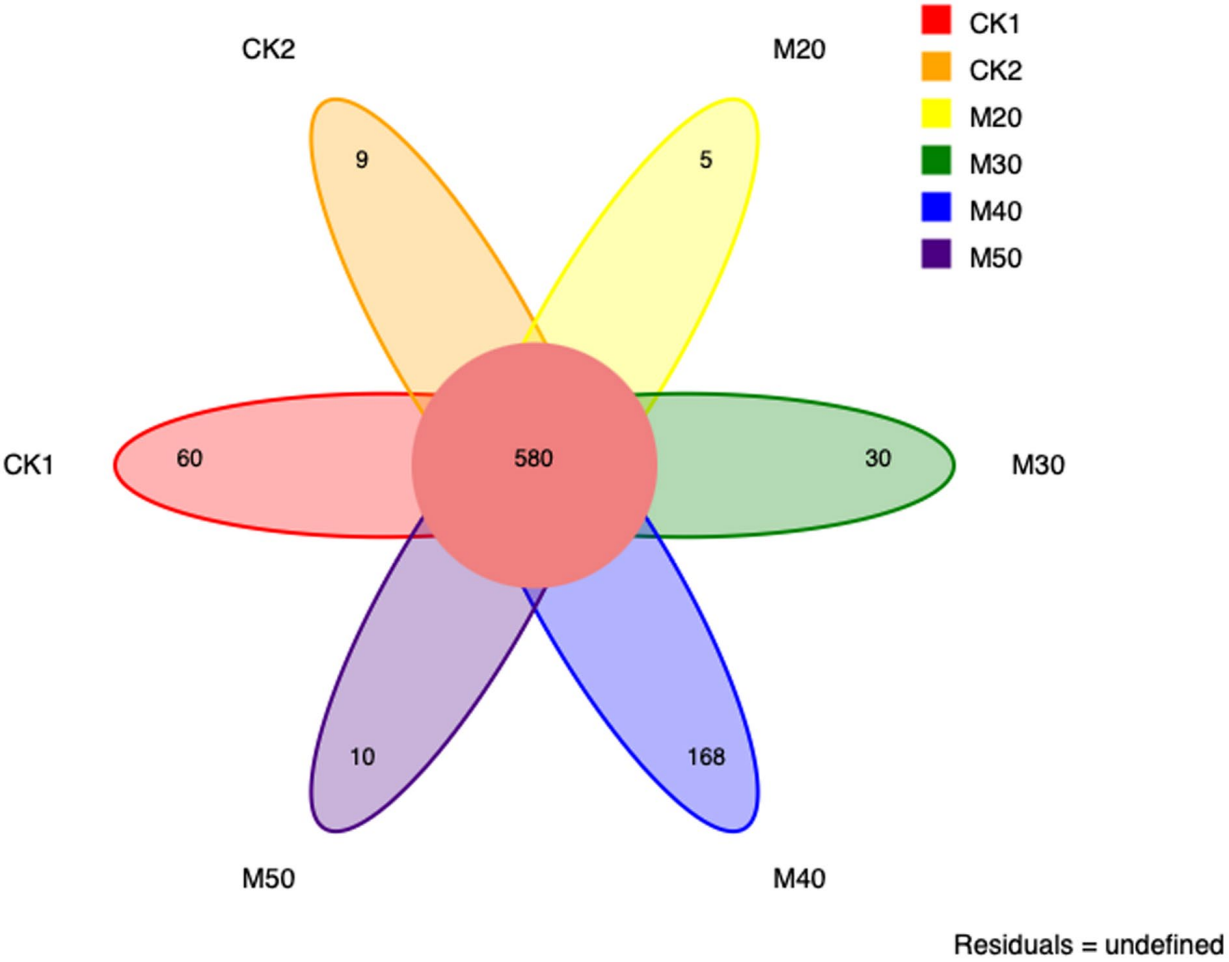
Numbers of unique OTUs of rumen bacteria for the different groups.

Cluster analysis using 16S rRNA gene sequences, based on a 97% similarity threshold, identified a total of 580 OTUs across all groups. The number of OTUs determined in each group varied: CK1 (60), CK2 (9), M20 (5), M30 (30), M40 (168), and M50 (10); the M40 group had the largest number of unique OTUs (Figure 1).

#### 3.1.2 Alpha diversity analysis of rumen bacteria from the different groups of sheep

According to Table 3, the coverage in all groups reached more than 99%, indicating a high coverage of rumen microorganisms. ACE and Chao1 indices were significantly lower in CK2 and M20 to M50 compared with CK1 (*p* < 0.05); there were no differences in the Shannon and Simpson indices between the groups (*p* > 0.05). For the Shannon index group, CK1 > CK2 > M40 > M20 > M50 > M30.

**Table 3:** Species richness and diversity indices of rumen bacteria.

Items	Ck1	Ck2	M20	M30	M40	M50	SEM	*p* value
Numbers of OTUs	640	589	585	610	748	590	15.20	0.581
ACE	1303^a^	1048^b^	1045^b^	908^c^	925^c^	852^c^	37.91	0.001
Chao1	1315^a^	1059^b^	1054^b^	911^bc^	935^bc^	861^d^	39.48	0.001
Simpson	0.02	0.04	0.02	0.04	0.03	0.04	0.003	0.402
Shannon	5.13	4.44	4.24	3.96	4.39	4.15	0.158	0.401
Coverage	99.6	99.7	99.7	99.6	99.7	99.8	0.019	0.315

In the same row, values with no letter or the same letter superscripts mean no significant difference (*P* > 0.05), while values with different superscripts are significantly different (*P* < 0.05).

#### 3.1.3 Rarefaction curves of rumen bacteria in sheep with different cotton straw levels

**Figure 2:**
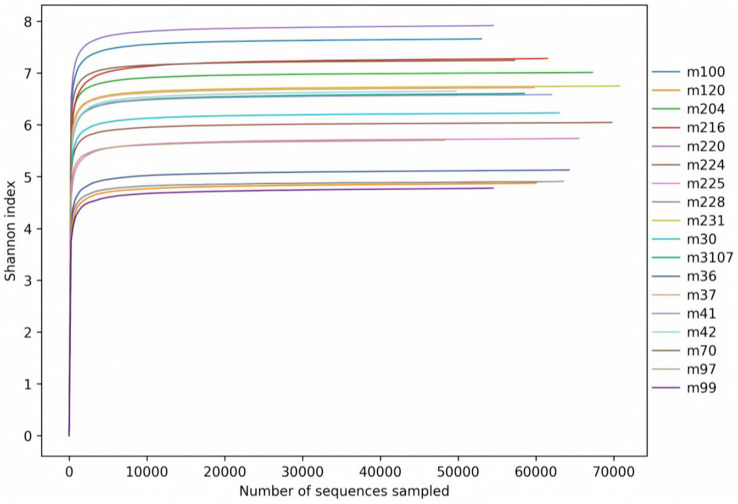
Shannon index rarefaction curves of rumen bacteria.

As can be seen from Figure 2, the Shannon index rarefaction curves for the bacteria of each group eventually flattened out, which means that the sequencing covered the vast majority of microorganisms in the samples.

#### 3.1.4 Principal component analysis (PCoA) of the effects of different cotton straw levels on the composition of rumen bacteria

**Figure 3:**
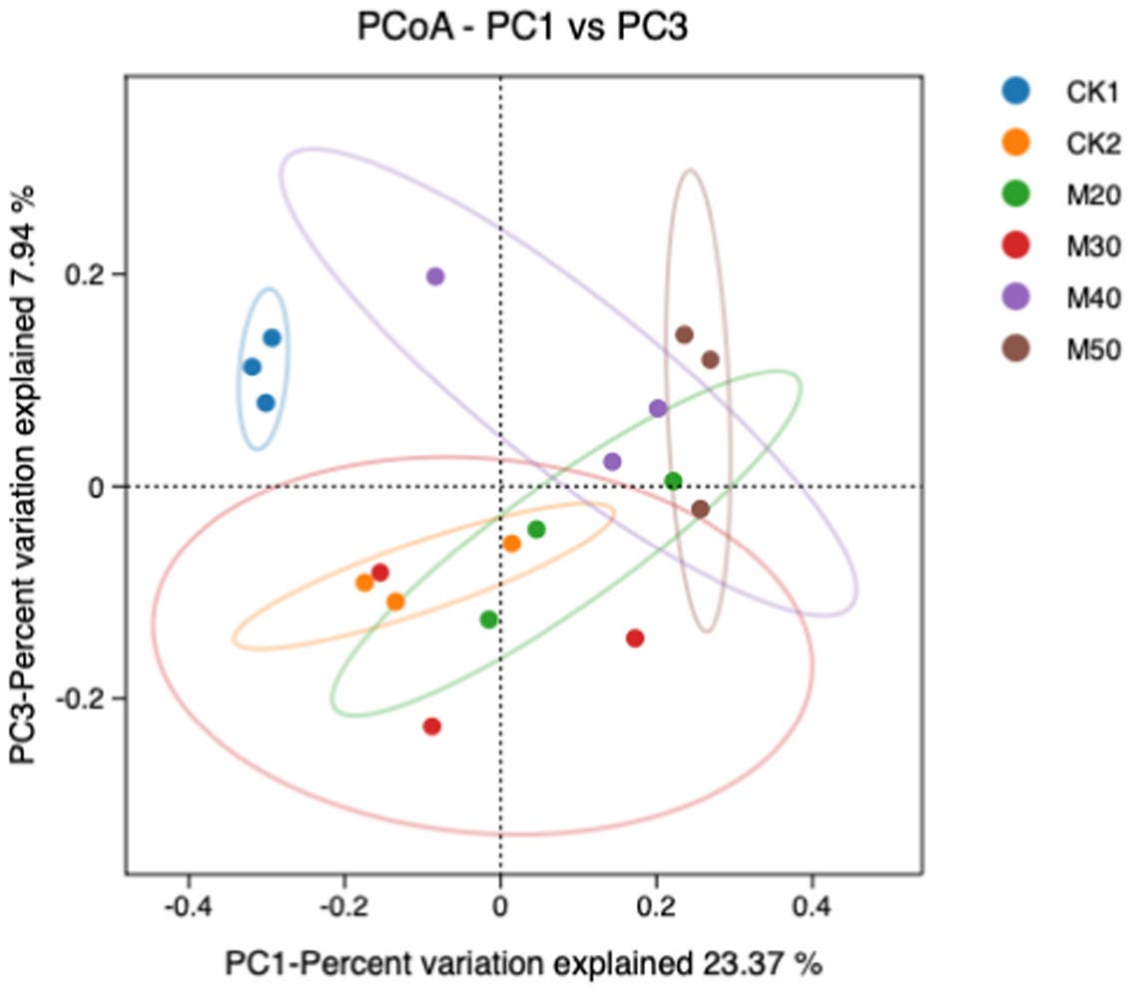
Principal component analysis (PCoA) of the effects of different cotton straw percentages on sheep rumen bacteria.

In Figure 3, PC1 is the 1st principal component, indicating 23.37% variance of isolated samples and PC3 is the 2nd principal component, indicating 7.94% variance of isolated samples. The compositions of the CK1 group, the CK2 group and the M50 group were significantly different from the CK2 group and the other test groups. The M20 group was not different from the CK2 group, but the M40 and M50 groups were significantly different from the CK2 group.

#### 3.1.5 Effect of different dietary cotton straw levels on the relative abundance of sheep rumen microbiota at the phylum level

**Figure 4:**
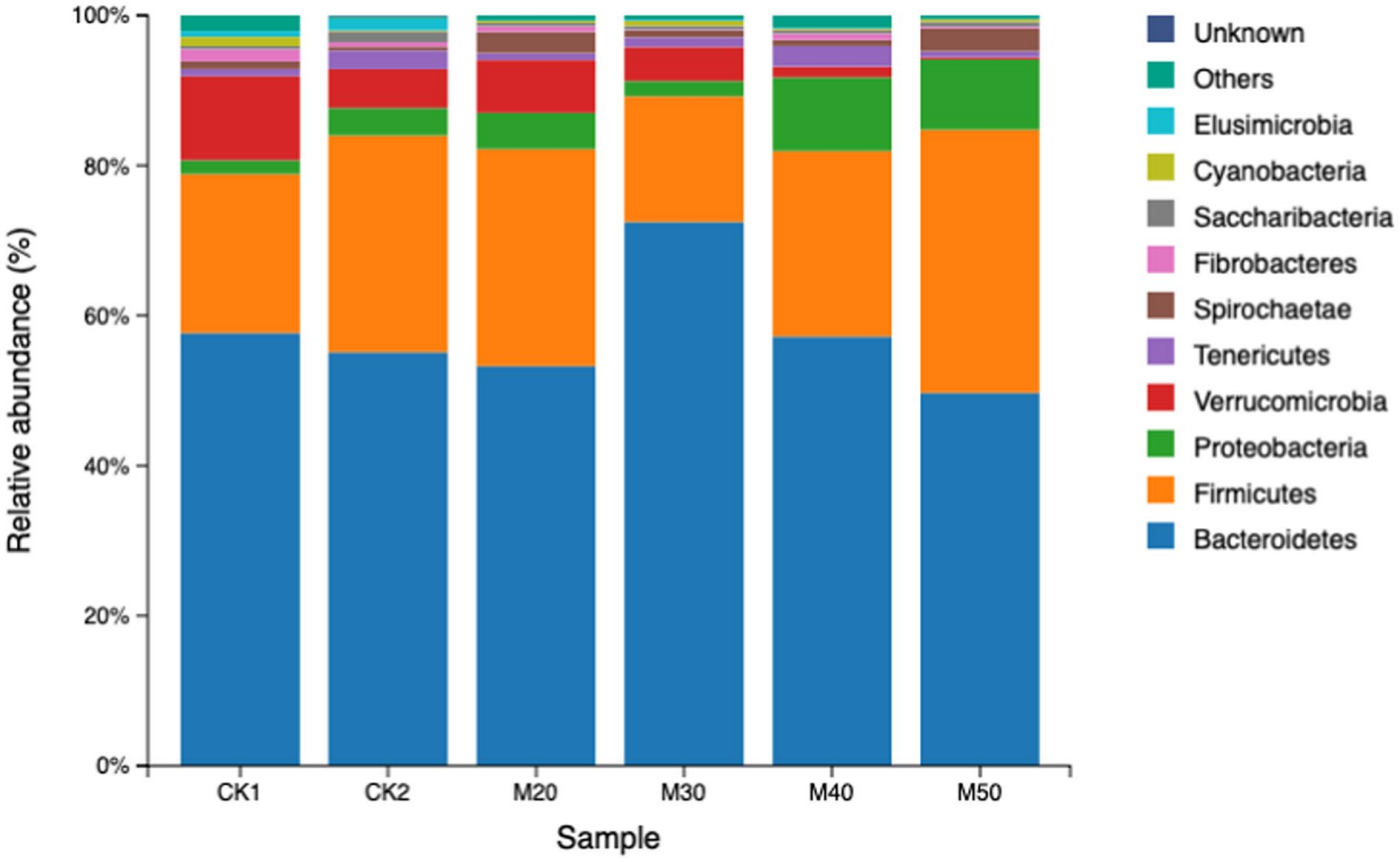
Relative abundance of rumen bacterial phyla in the various groups.

The results are shown in Table 4. The difference in relative abundance of Bacteroidetes and Firmicutes in the test groups was not significant (*p* > 0.05). The Proteobacteria abundance in the M40 and M50 groups was significantly higher than that of the CK1 group (*p* < 0.05). The abundance of Verrucomicrobia gradually decreased with increasing cotton straw content (*p* > 0.05). The differences in abundance of Firmicutes, Spiroplasma and fiber bacteria were not significant between the groups (*p* > 0.05). The Elusimicrobia abundance in each test group was significantly lower than that of the CK2 group (*p* < 0.05). Except for the M30 group, the abundance of Cyanobacteria in the CK2 group and each test group was significantly lower than that of the CK1 group (see Figure 4).

**Table 4:** Effect of feeding different percentages of cotton straw on the abundance of rumen bacteria at the phylum level.

Phylum	Ck1	Ck2	M20	M30	M40	M50	SEM	*P value*
Bacteroidetes	57.67	55.11	53.25	72.49	57.19	49.70	3.35	0.547
Firmicutes	21.18	28.86	28.91	16.69	24.72	35.03	2.82	0.579
Proteobacteria	1.84^b^	3.66^ab^	4.81^ab^	2.04^b^	9.80^a^	9.40^a^	1.07	0.04
Verrucomicrobia	11.18	5.21	7.08	4.59	1.47	0.28	1.39	0.216
Tenericutes	0.99	2.48	0.91	1.29	2.74	0.80	0.31	0.275
Spirochaetae	1.01	0.50	2.84	0.87	0.81	3.03	0.48	0.549
Fibrobacterota	1.64	0.59	0.75	0.13	0.85	0.24	0.20	0.358
Saccharibacteria	0.39	1.44	0.43	0.50	0.49	0.62	0.12	0.085
Cyanobacteria	1.24^a^	0.15^b^	0.27^b^	0.63^ab^	0.14^b^	0.33^b^	0.12	0.02
Elusimicrobiota	0.76^ab^	1.53^a^	0.10^b^	0.16^b^	0.088^b^	0.067^b^	0.18	0.035

In the same row, values with no letter or the same letter superscripts mean no significant difference (*P* > 0.05), while values with different superscripts are significantly different (*P* < 0.05).

#### 3.1.6 Effect of different dietary cotton straw levels on the relative abundance of sheep rumen bacteria at the genus level

**Figure 5:**
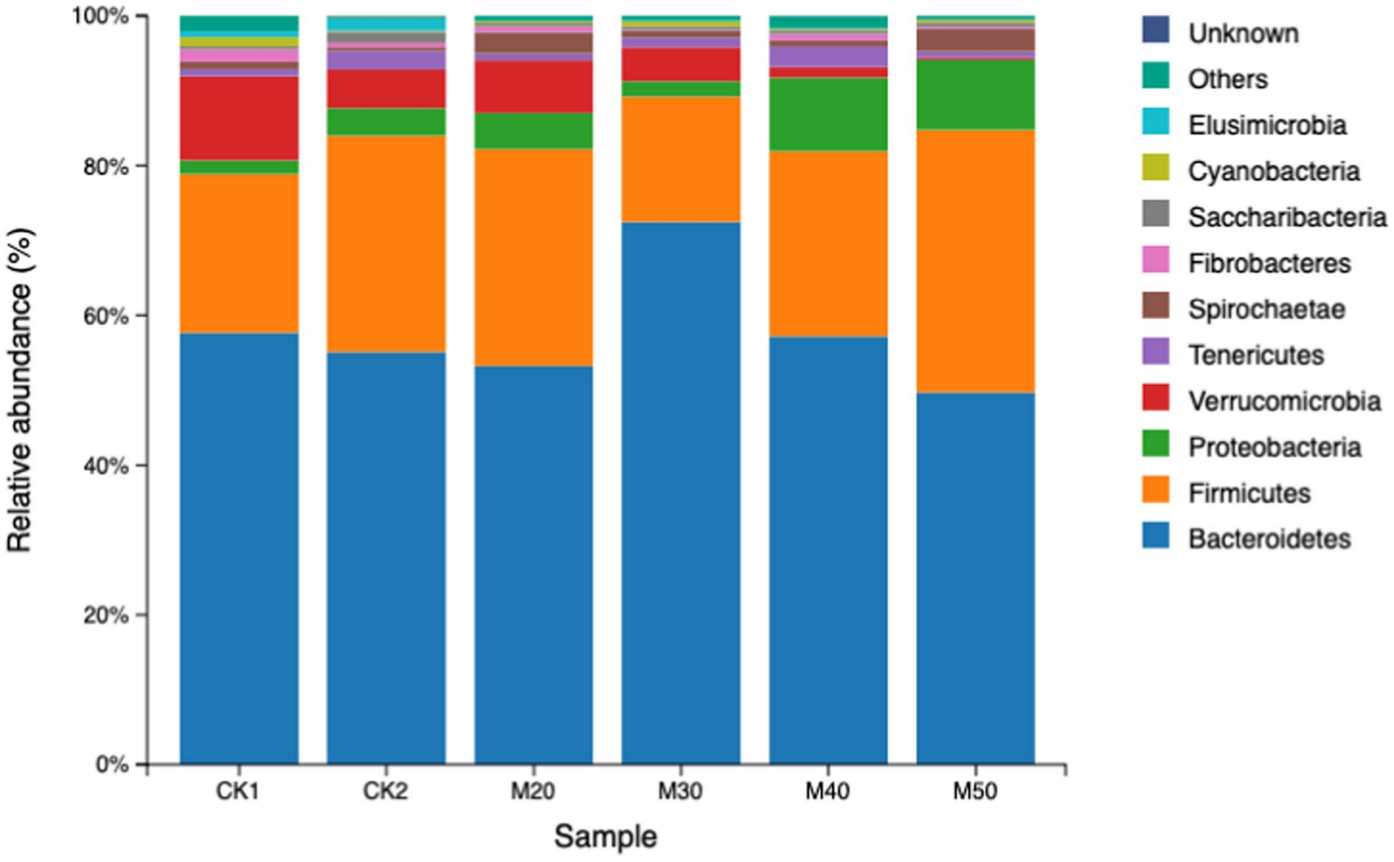
Relative abundance of rumen bacterial taxa in the various groups.

The data are shown in Table 5. *Prevotella_1* abundance in the CK2 and M20-M50 was higher than that in the CK1(*p* > 0.05). *Rikenellaceae_RC9_gut_group* in the M20 and M40 was lower than in the CK2 (*p* > 0.05). The abundance of *Ruminococcaceae_UCG-002* was lower than that in the CK1 group (*p* < 0.05). The *Ruminococcus_1* in the CK2, M20, and M40 groups was significantly higher than that in the CK1 group (*p* < 0.05) (Figure 5).

**Table 5:** Effect of feeding different proportions of cotton straw on the abundance of rumen bacteria at the genus level.

Taxa	Ck1	Ck2	M20	M30	M40	M50	*SEM*	*P* value
*Prevotella_1*	18.9	30.0	39.4	42.3	24.0	30.9	3.41	0.38
*Uncultured rumen bacteria*	24.44	8.77	11.14	11.32	10.51	4.25	1.98	0.051
*Rikenellaceae_RC9_gut_ group*	12.28	13.19	2.08	11.97	3.24	7.01	1.52	0.085
*Prevotellaceae_UCG-001*	1.92	4.27	3.55	5.72	8.07	4.73	1.01	0.689
Unclassified	6.09	3.83	2.30	2.92	3.53	3.88	0.50	0.359
*Prevotellaceae_UCG-003*	5.14	0.86	1.63	2.09	7.07	0.65	1.11	0.527
*Succiniclasticum*	0.88	0.69	2.67	0.75	4.77	1.51	0.72	0.580
*Erysipelotrichaceae _ UCG-004*	1.11	5.18	2.91	0.60	0.56	0.23	0.77	0.408
*Treponema_2*	0.92	0.48	2.83	0.83	0.76	3.00	0.48	0.583
*Selenomonas_1*	0.62	0.29	2.37	0.24	2.91	2.35	0.51	0.556
*Ruminococcaceae _ UCG- 002*	4.44^A^	0.21^B^	0.60^B^	1.20^B^	0.11^B^	0.04^B^	0.408	0.000
*Ruminococcus_1*	0.29^b^	1.61^a^	1.47^a^	0.62^ab^	1.2^a^	0.44^b^	0.162	0.04

In the same row, values with different lowercase superscript letters differ significantly at P <0.05; values with different uppercase superscript letters differ significantly at *P* <0.01. Values sharing the same letter, or without a superscript, are not significantly different.

#### 3.1.7 Linear discriminant analysis effect size (LEfSe) of bacterial composition of sheep rumen

**Figure 6:**
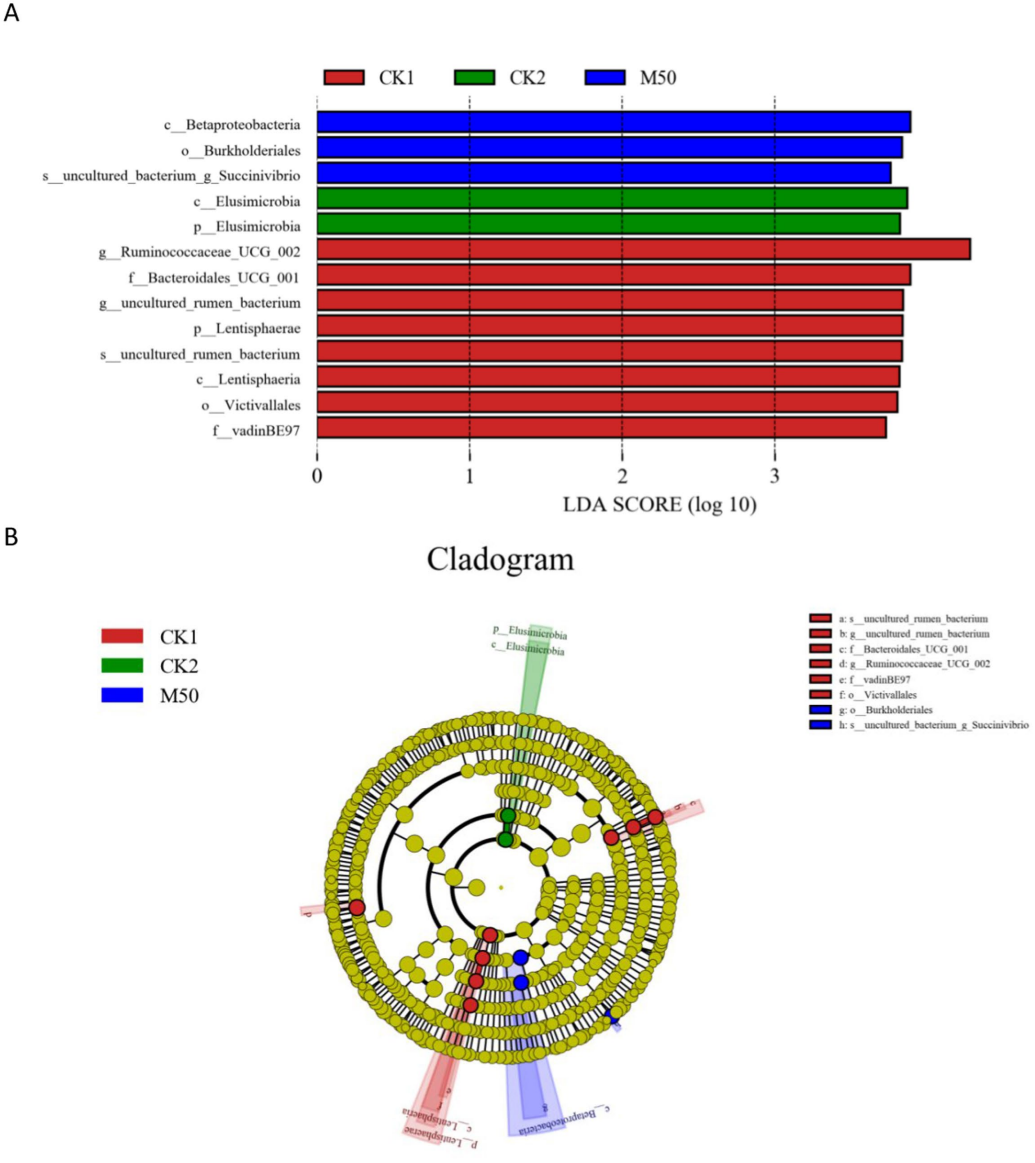
**(A)** Bacterial taxa with the highest LDA scores in the CK1, CK2 and M50 groups. **(B)** Cladogram of bacterial taxa for CK1, CK2 and M50.

This section describes the determination of rumen bacterial composition in sheep fed different percentages of cotton straw added to the basic diet. Figure 6A presents the results of a differential bacterial analysis. Linear discriminant analysis (LDA) identified 13 bacterial species with significant differences across the dietary groups, each with LDA scores >3.0. Among these species, eight exhibited especially high LDA scores, predominantly in the CK1 group. Three species showed strong associations with the M50 group and two with the CK2 group. Figure 6B depicts the phylogenetic relationships of these 13 species in the evolutionary clade. Using LEfse analysis of ITS sequencing data, we identified characteristic microorganisms in each group. The CK1 group is characterized by six taxa: family Ruminococcaceae-UCG-002, phylum Bacteroidetes-UGG-001 (uncultured tumor bacterium), Lentisphaeria (uncultured tumor bacterium, family Lentisphaeraceae, class Victivallales, family vadin-BE 97). The characteristic bacterial taxa of CK2 are phylum Elusimicrobiota and class Elusimicrobia. The characteristic bacterial taxa of the M50 group are class betaproteobacteria, order Burkholderiales, and uncultured bacterial genus *Succinivibrio*.

### 3.2 Analysis of the rumen fungal microbiota in sheep

#### 3.2.1 Number and specificity of rumen fungal OTUs from sheep with different percentages of cotton straw in feed

**Figure 7:**
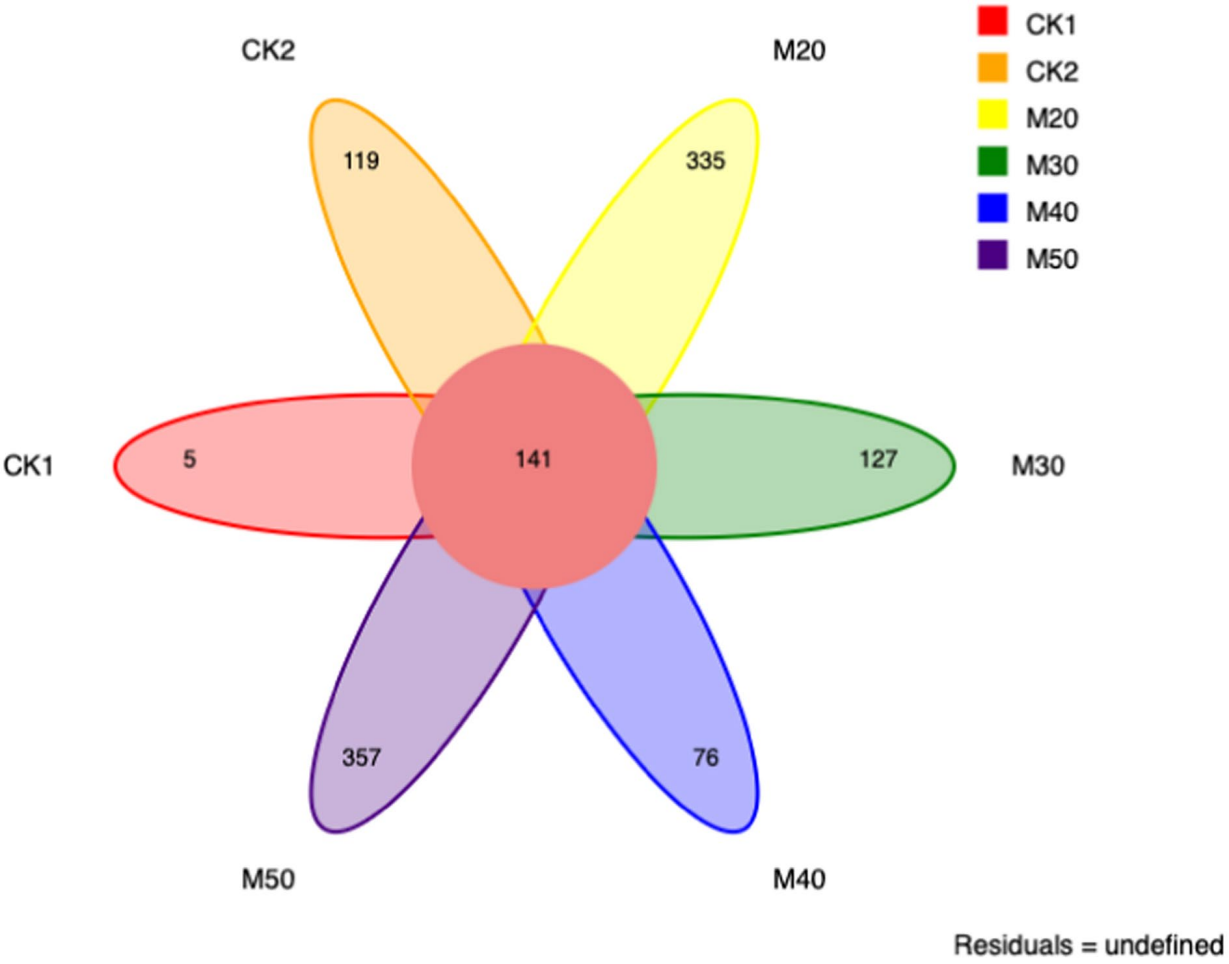
OTUs of ruminal fungi from each dietary group.

As shown in Figure 7, the number of unique OTUs varied from group to group with the M20 and M50 groups having the largest number: CK1 (5), CK2 (119), M20 (335), M30 (127), M40 (76) and M50 (357).

#### 3.2.2 Alpha diversity analysis of rumen fungi from sheep with feed containing different cotton straw levels

As shown in Table 6, the coverage in each group was >99%. The ACE index, Chao1 index, Shannon index, and Simpson index were not significantly different (*p* > 0.05).

**Table 6:** Richness and diversity indices of ruminal fungal species.

Items	Ck1	Ck2	M20	M30	M40	M50	SEM	*P* value
OTU quantity	290	317	778	355	321.67	703	334.47	0.691
ACE	660	349	819	367	337	721	195.14	0.810
Chao1	474	359	829	369	345.19	724	821.37	0.784
Simpson	0.24	0.32	0.14	0.15	0.26	0.25	54.99	0.913
Shannon	2.03	2.80	3.48	3.13	2.42	3.34	19.50	0.868
Coverage	99.8	99.9	99.8	99.9	99.9	99.9	0.011	0.502

#### 3.2.3 Shannon index rarefaction curves of rumen fungi from sheep fed with different cotton straw amounts

**Figure 8:**
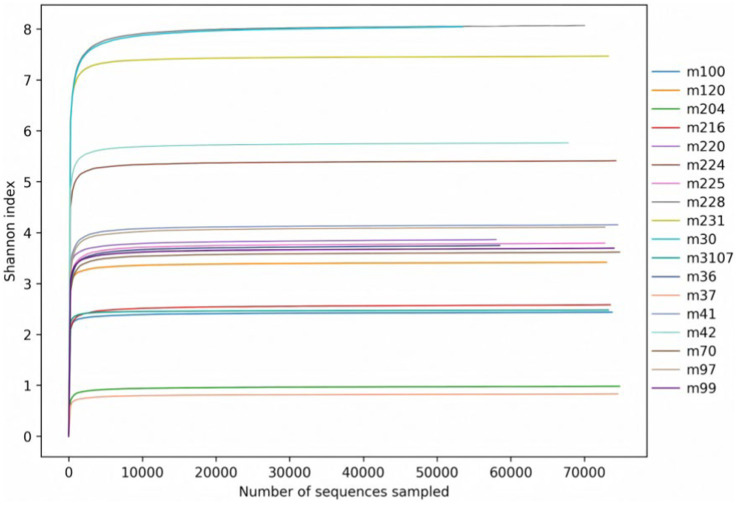
Rarefaction curves for Shannon indices vs. number of rumen fungi sampled.

As can be seen in Figure 8, the fungal Shannon index rarefaction curves of each group eventually plateaued, which means that the sequencing covered the majority of microorganisms in the samples.

#### 3.2.4 Principal component analysis (PCoA) of the effects of different cotton straw levels on the sheep rumen fungal population

**Figure 9:**
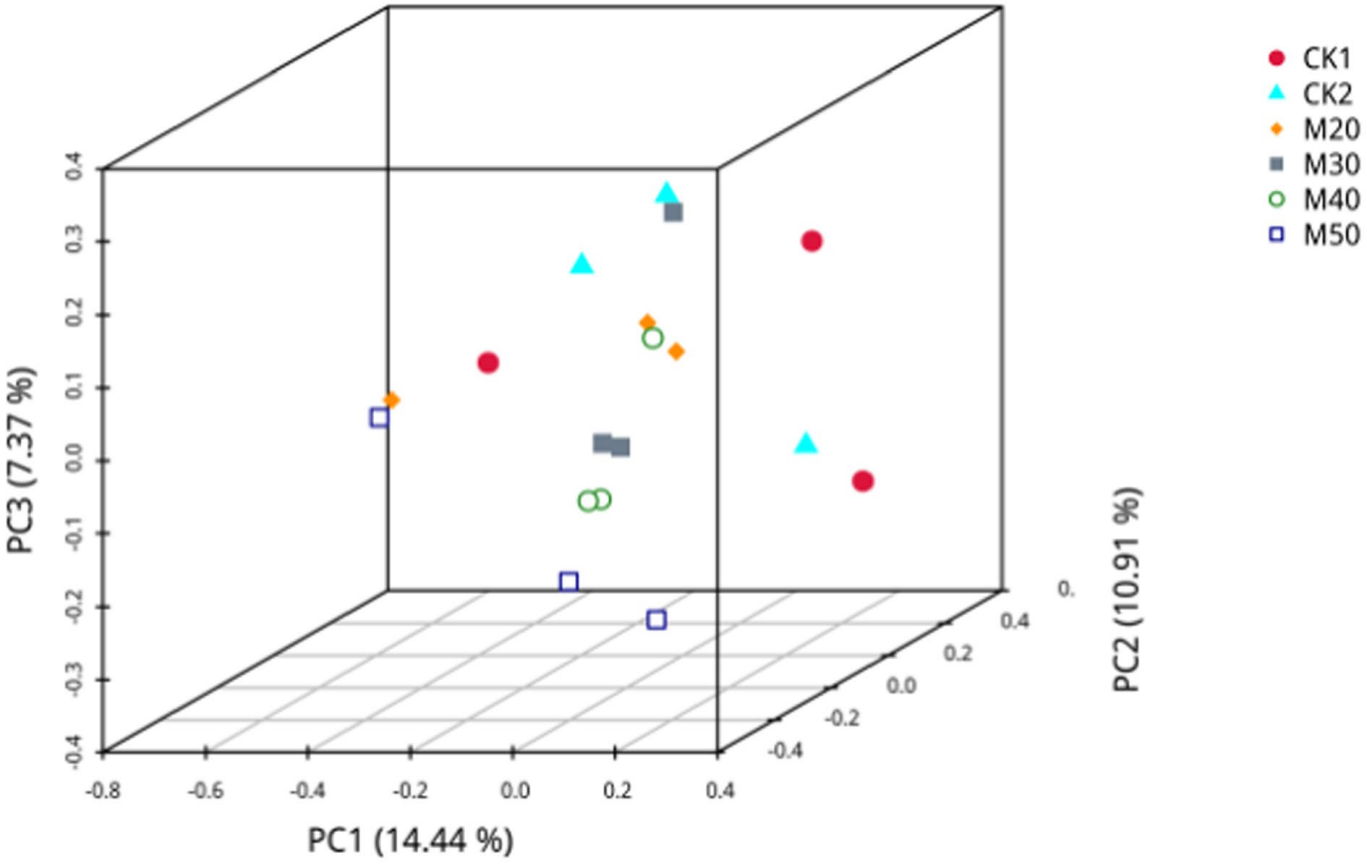
Principal component analysis of the effects of different amounts of cotton straw on the population of rumen fungi in sheep.

As shown in Figure 9, PC1 is the 1st principal component, covering 14.44% of the variance of the isolated samples, PC2 is the 2nd principal component, representing 10.91% of the variance of the isolated samples, and PC3 is the third principal component, accounting for 7.34% of the variance of the isolated samples. Using the Binary-Jaccard unweighted distance algorithm, the different color points represent samples in each group. The further apart two samples are, the larger the significant difference in species composition, and the closer they are, the more similar is the species composition. The larger sample point spacing between the M50 and CK2 groups indicates a greater difference in fungal composition between the two groups.

#### 3.2.5 Effect of different cotton straw percentages in the feed on the relative abundance of rumen fungi

**Figure 10:**
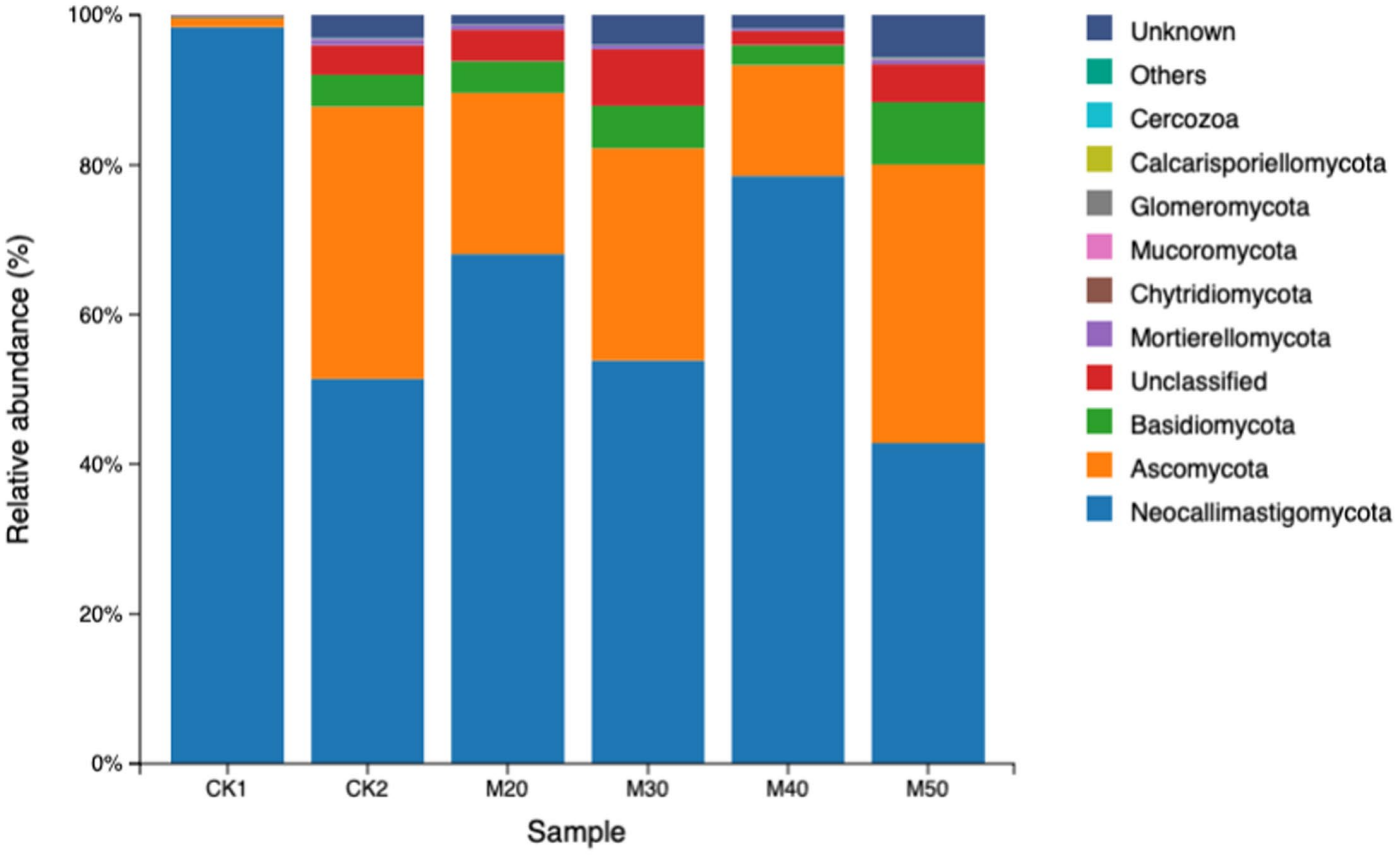
Relative abundance of rumen fungal phyla in the various groups.

As shown in Table 7, differences in the relative abundance of fungi in each group were not significant. In the CK1 group, the phylum Neocallimastigo-mycota accounted for more than 98%, while in groups CK2 and M20-M50, the main fungal phylum was also Neocallimastigo-mycota (42.08 to 78.56%). The Ascomycota accounted for 14.66 to 37.76%, the Basidiomycota 2.65 to 8.43%, and unclassified fungi 1.97 to 7.73% (see Figure 10)

**Table 7:** Effects of different feed percentages of cotton straw on the relative abundance of fungal phyla in sheep rumen.

Items	Ck1	Ck2	M20	M30	M40	M50	SEM	*P* value
*Neocallimastigo-mycota*	98.1	51.1	63.6	52.8	78.6	42.1	8.19	0.412
*Ascomycota*	1.3	36.6	24.8	28.9	14.7	37.8	5.61	0.435
*Basidiomycota*	0.20	4.25	4.68	5.76	2.65	8.43	1.05	0.349
*Unclassified*	0.20	4.01	4.74	7.73	1.97	5.15	1.01	0.378
*Mortierellomycota*	0.02	0.75	0.79	0.51	0.25	0.62	0.14	0.644

#### 3.2.6 Effect of different dietary cotton stalk levels on the relative abundance of sheep rumen fungi at the genus level

**Figure 11:**
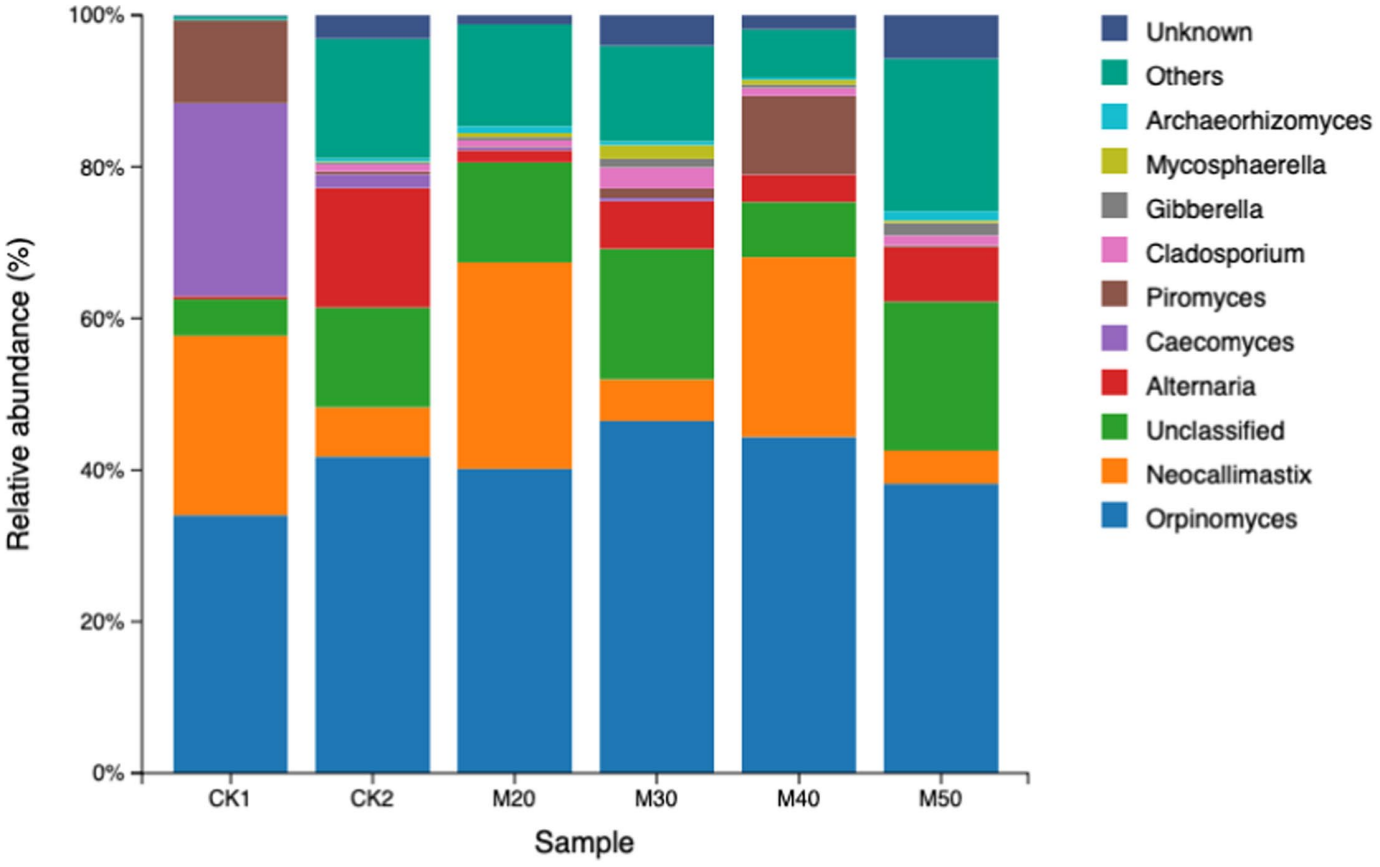
Relative abundance of rumen fungal taxa in the various groups.

As shown in Table 8, *Caecomyces* and *Piromyces* in the CK2 group and M20-M50 groups decreased, but the difference was not significant (*p* > 0.05), while *Alternaria* increased compared with the CK1 group. *Cladosporium* and *Mycosphaerella* in the M30 group were higher than in the CK1 group (*p >* 0.05), and that in the M50 group was higher than in the CK1 group (*p >* 0.05) (see Figure 11).

**Table 8:** Effect of different percentages of cotton straw in feed on the relative abundance of rumen fungal taxa in sheep.

Taxa	Ck1	Ck2	M20	M30	M40	M50	SEM	*P* value
*Orpinomyces*	33.3	41.4	37.6	45.5	45.8	37.3	8.01	0.998
*Neocallimastix*	25.8	6.5	25.3	5.4	22.5	4.6	4.94	0.646
Unclassified fungal genus	5.09	13.19	15.17	17.62	7.37	19.94	2.87	0.701
*Alternaria*	0.45	15.92	1.56	6.41	3.52	7.17	2.31	0.475
*Caecomyces*	24.05	1.80	0.42	0.42	0.02	0.00	3.34	0.222
*Piromyces*	10.41	0.37	0.10	1.26	10.17	0.22	1.62	0.119
*Cladosporium*	0.05	0.98	0.92	2.82	1.02	1.30	0.32	0.233
*Gibberella*	0.04	0.24	0.47	1.16	0.44	1.67	0.20	0.141
*Archaeorhizomyces*	0.03	0.421	1.09	0.51	0.31	1.27	0.21	0.558
*Mycosphaerella*	0.02	0.11	0.52	1.86	0.62	0.31	0.23	0.183

#### 3.2.7 Linear discriminant analysis effect size (LEfSe) analysis of sheep

**Figure 12:**
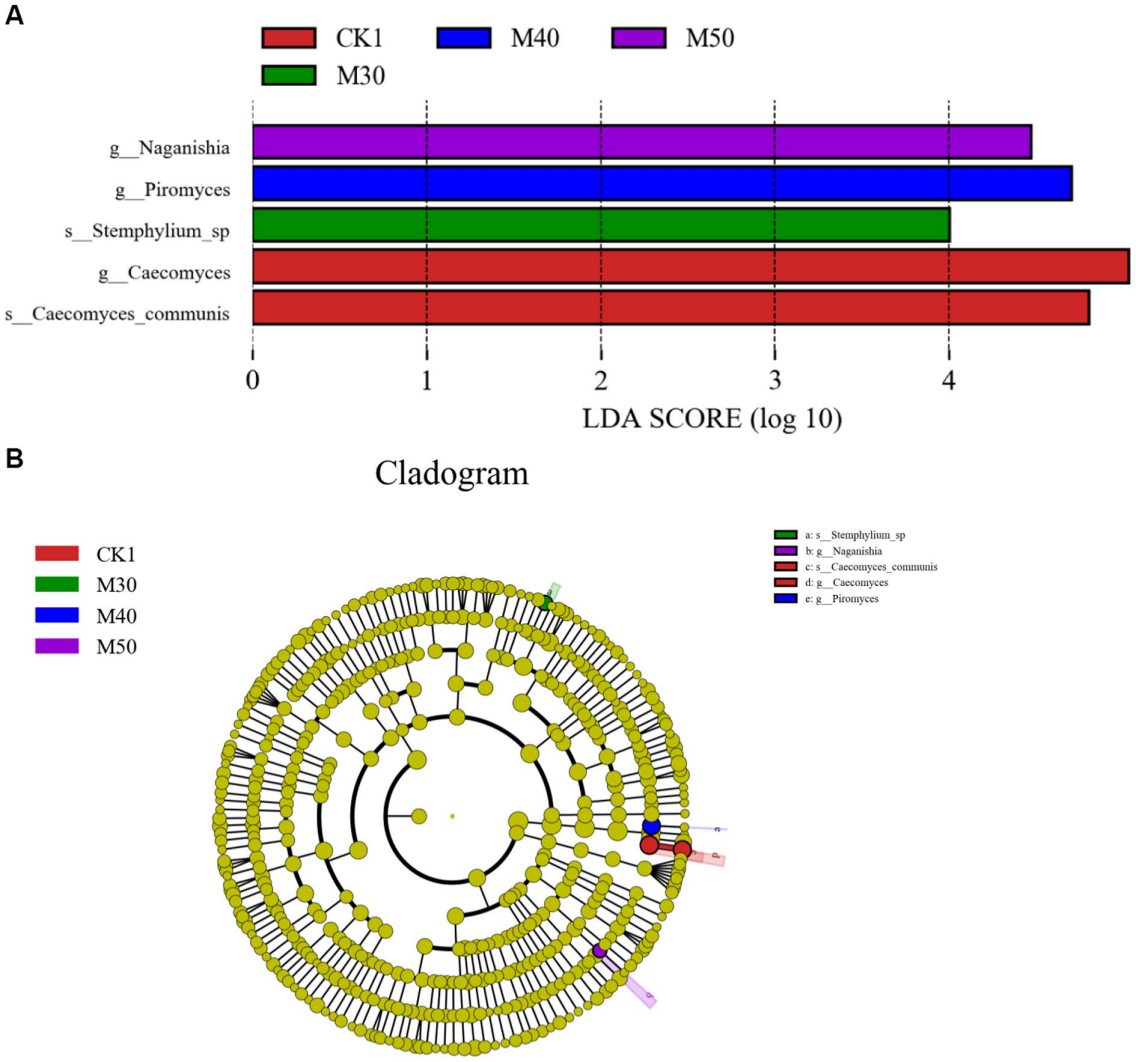
**(A)** LDA scores of the predominant rumen fungi from groups CK1 and M30-50. **(B)** Cladogram depicting phylogenetic relationships between the five most abundant fungal taxa in the rumen.

The results of the LEfSe analysis are shown in Figure 12A. LDA, five divergent species with LDA values >4.0, including two species with especially high LDA scores. The CK1 group and the M30, M40 and M50 groups each had relatively high LDA scores, and the five species in the evolutionary clade are shown in Figure 12B. The two characteristic genera in the CK1 group were g-*Caecomyces* and s-*Caecomyes*-communis. The characteristic fungi in the M30 group were s-*Stemphylium*-sp., the characteristic fungus in the M40 group was g-Piromyces, and the characteristic fungal genus in M30 group was g-*Naganishia*.

## 4 Discussion

Ruminal microorganisms are mainly composed of bacteria, fungi, archaea and protozoa. The main function of rumen bacteria is to metabolize cellulose and pectin to produce formic acid and acetic acid, and also to break down starches and sugars. In contrast, the rumen fungi assist in the digestion of fiber, converting it into nutrients that animals can take up. They also participate in the degradation of proteins.

### 4.1 Effect of different amounts of cotton straw added to feed on the rumen bacteria of sheep

The number of OTUs represents the number of species with greater than 97% sequence similarity to rumen microbiota, and one of the main factors affecting the number of microbial species is diet. Unlike corn straw and wheat straw, cotton straw contains free gossypol and lignin which can affect feed utilization by ruminants. Zhao et al. added free gossypol and gossypol acetate to feed and found that the number of rumen bacterial OTUs in the free gossypol group was lower than that of the gossypol acetate and control groups (Zhao, 2023). Free gossypol above a certain concentration is toxic and can damage microbial cells by disrupting the structure and function of the cell membranes and inhibiting metabolic activities. This toxic effect can impair the growth of some microorganisms or even cause death, thereby reducing the number of OTUs. In our study, except for the M40 group, the number of OTUs in the CK2 and other groups was lower than that in the CK1 group. The M40 group had the largest number of OTUs for free gossypol below the safety threshold (<200 mg/kg), apparently because of the development of tolerance to free gossypol.

Shabat et al. (2016) found that the richness of microbial 16S rRNA was negatively correlated with feed conversion efficiency. We used the ACE and Chao indices to estimate unobserved species richness and found that the rumen bacterial ACE was significantly reduced, similar to the findings of Li et al. (2020). The addition of cotton straw to the feed increased the abundance of fiber-degrading rumen bacteria (Hou, 2013), probably because gossypol has a growth-promoting effect on rumen fiber-degrading bacteria. The Shannon index measures the species diversity of the biome, and the higher the value, the larger the diversity. The Simpson index shows the opposite effect. The difference between the Shannon index and the Simpson index in this experiment was not significant, indicating that the addition of cotton straw had no significant effect on the diversity of the rumen bacterial diversity.

Beta diversity analysis is commonly used to determine the biodiversity and microbial community composition among different samples. Zhao (2023) investigated rumen fermentation in sheep fed a diet containing cottonseed shells and cottonseed meal containing free gossypol. They observed significant differences in PCA between the cottonseed group and the control group without addition. Li et al. (2020) showed that there were differences in the microbial community composition between 100% replacement of green hay and 50% replacement and control groups. Kaixin (2023) reported that adding 14.5 to 29% cotton leaves and fermented cotton stalks to a sheep-fattening diet affected *β*-diversity relative to the control group. The results are consistent with the differences in microbial community composition between the M50 and CK1 and CK2 groups seen in this study. Thus, the addition of cotton straw to the diet can change the composition of the microbial community of sheep rumen bacteria, and the reason may be related to the increasing content of free gossypol or lignin, although the free gossypol content is within the allowable range.

Studies have shown that Bacteroidetes, Firmicutes, and Proteobacteria are the dominant bacterial phyla in the rumen of ruminants (Malmuthuge et al., 2014; Liu et al., 2017; Yuan et al., 2018). Adding specific compounds to the feed to regulate the rumen microbiota can effectively improve the feed conversion efficiency and growth performance of sheep, and the core microorganisms such as rumen Bacteroidetes and Firmicutes play a leading role (Lu et al., 2021). Proteobacteria is a phylum mainly containing gram-negative bacteria, including several pathogens such as *Escherichia coli*, *Salmonella*, *Helicobacter pylori*, etc. (Brock, 2000). It also includes many bacteria responsible for nitrogen fixation (Moulin et al., 2001).

Verrucomicrobia can produce short-chain fatty acids and promote mucin degradation. The dominant ruminal bacteria in each group were Bacteroidetes and Firmicutes, which is consistent with the present study. The addition of cotton straw to the M40 and M50 groups significantly increased the abundance of Proteobacteria. This change is usually characterized by a decrease in the relative abundance of other phyla. The M50 group had significantly reduced abundance of Verrucomicrobia, which differs from the findings of Li and Qi (Li et al., 2020). Additionally, some genera within the Proteobacteria phylum may be involved in the fixation and utilization of ammonia nitrogen. For example, *Nitrosomonas, Nitrosospiracertai*, *Pseudomonas*, and *Rhizobium* can convert ammonia nitrogen into organic nitrogen, thereby affecting the nitrogen cycle in the rumen. However, the Proteobacteria phylum contains many opportunistic pathogens (such as *Escherichia coli* and *Salmonella*), and an increase in its abundance may raise the risk of infection in the host. Kaixin (2023) showed no significant change in ruminal Proteobacteria, but Zhao (2023) found that adding free gossypol increased the abundance of Proteobacteria and reduced Verrucomicrobia. This also indicates that the addition of more than 40% cotton straw carries a potential risk for rumen health and the production of short chain fatty acids. Some cyanobacteria can fix nitrogen under anaerobic conditions and convert it into NH_3_, NO_2_^−^, and NO3-for use by host cells Golden and Yoon (1998). In our study, except for the M30 group, the abundance of cyanobacteria was significantly reduced compared with the CK1 group similar to the data of Zhao (2023). The addition of free gossypol increased the abundance of cyanobacteria (Li et al., 2020), which may be because of differences in the protein component of the feed.

*The Prevotella_*1 genus is a class of strictly anaerobic polyform bacteria, widely distributed in the rumen. It plays an important role in the digestion of hemicellulose, non-fibrous carbohydrates, and proteins, and it can also degrade plant cell walls (Kabel et al., 2011; Bekele et al., 2010). The *Prevotellaceae* UCG-001 genus is a beneficial family of bacteria with anti-inflammatory properties and the ability to alleviate disturbances in glucose and lipid metabolism. In our experiments, the abundance of the *Prevotella_1* genus and *Prevotellaceae UCG-001* genus in the cotton straw groups was higher than in the CK1 group. The difference was similar to the results of Kaixin (2023) with the addition of micro-scale cotton stalks, suggesting that increasing the content of cotton stalks in feed enhances rumen fiber degradation. The *Rikenellaceae_RC9_gut_group* contains genera of butyrate-producing bacteria, which is important for maintaining intestinal SCFA levels, and may regulate fat deposition by affecting the concentration of volatile fatty acids (Cheng et al., 2022). The abundance of the *Rikenellaceae_RC9_gut_group* in the M20 and M40 groups was lower than that in the CK2 group in agreement with Kaixin (2023). The results of microbial fermentation of added cotton stalks were similar, indicating that the addition was not conducive to butyrate production. The abundance of *Ruminococcaceae UCG-002* was significantly negatively associated with many indicators of chronic metabolic diseases, which may affect disease development by modulating the metabolism of secondary bile acids such as isolithocholic acid (Jiang et al., 2022). In our investigation, the *Ruminococcaceae UCG-002* genera were significantly decreased with increasing content of cotton straw, indicating that the addition may affect bile acid metabolism. *Ruminococcus* is a genus of bacteria producing abundant propionic and butyric acid, which plays an important role in the digestion of resistant starch and fiber (Tyakht et al., 2013; Reichardt et al., 2014). In our experiments, the abundance of *Ruminococcus_1* in the CK2, M20 and M40 groups was higher than that of the CK1 group, and the dietary lignin content increased with increasing cotton straw content, which promoted the proliferation of *Ruminococcus_1* in response to the high fiber diet and facilitated the digestion of roughage.

### 4.2 Effect of increasing cotton straw content on the sheep rumen fungi

Related studies have shown that ruminant fungi are eukaryotic microorganisms that influence nutrient absorption by osmotic effects (Voigt et al., 2021). Anaerobic fungi in the rumen are involved in the digestion of lignocellulose (Ravi et al., 2021; Cheng et al., 2018). The number of fungal OTUs is influenced by the dietary fiber content and the rumen microbiota. When anaerobic fungi are removed from the rumen, sheep reduce their feed intake by 40%, but it can also affect dry material digestibility and milk production (Guo et al., 2020). The addition of 0.1% gossypol to the sheep diet significantly reduced the fungal population (Hou, 2013). There were 141 OTUs, and the M20 and M50 groups had the largest number of OTUs. The Chao1 and ACE indices were the highest, but the difference was not significant. Beta diversity analysis and PCoA revealed the fungal diversity of each group, and the distance between each group was not very large, indicating that the different percentages of cotton straw affected the species and biodiversity of fungi in the rumen. This may be related to the difference of neutral detergent fiber content in each group in this experiment.

The rumen fungi, which require a strictly anaerobic environment for growth, play an important role in the degradation of fiber in the diet, and can also degrade starch, glycogen and other substances (Zhang et al., 2022). The Neocallimastix phylum is one of the commonest microorganisms in the digestive tract of lactating herbivores, and plays a crucial role in the degradation of woody and fibrous feed materials (Wang et al., 2019). The Ascomycota phylum is the largest phylum in the fungal kingdom, and its taxa possesses a variety of highly active carbohydrate-degrading enzymes that function in various nutrient cycles. They cause mildews of wood, food, cloth and leather and the decomposition of animal and plant residues (Luo et al., 2021). The Basidiomycota phylum not only produces unique small and medium-sized molecular metabolites, such as phenazine, volatile flavors, azo pigments, antibiotics and glucose, but also secrete a set of specific lignin-digesting enzymes that enable them to thrive on lignocellulose, the phylum contains some pathogenic taxa like *Stachybotrys*, but also many medicinal species such as *Ganoderma lucidum* and antitumor fungi (Roger et al., 1992; Ho et al., 2000).

Fan et al. (2024) in a study of the influence of diet on the dominant rumen fungi of beef cattle, identified the Ascomycota phylum, Basidiomycota phylum, and Neocallimastix phylum. Xu et al. (2020) fed cows a refined coarse fiber diet and identified Ascomycota phylum and Neocallimastix phylum as the dominant fungi phylum. In this study, the relative abundance of phylum Neocallimastix in the CK1 group was 98%, followed by phylum Ascomycota at 1.29%. The phylum Basidiomycota, the unclassified bacteria phyla, and the silk aria phyla accounted for no more than 1%. In the CK2 group as well as in the M20-M50 groups, the Neocallimastix and Ascomycota were also the dominant fungal phyla. The abundance of Neocallimastix in the rumen accounted for 42.08–78.56% of the fungi, while the Ascomycota made up 14.66–37.76%, the Basidiomycota represented 2.65 to 8.43%, and unclassified phyla comprised 1.97 to 7.43%. This suggested that the relative abundance of the dominant phyla in the different cotton straw groups was altered through addition of the lignin-containing roughage, which increased the abundance of fungal phyla that degrade lignin.

*Orpinomyces* genus can secrete plant cell wall-degrading enzymes, facilitating the degradation and transformation of fiber substances (Abhaya et al., 2017). *Lamytridiae* genus and *Neocallimastix* genus in the rumen degrade lignocellulose more efficiently (Fliegerova et al., 2015), while *Caecomyces* genus is also involved in the degradation of lignocellulose. From our results, the dominant fungal genera were: (1) CK1 group, *Orpinomyces* genus, *Neocallimastix* genus, *Caecomyces* genus, and *Piromyces* genus; (2) CK2 group, *Orpinomyces* genus, *Neocallimastix* genus, and *Alternaria* genus; (3) M20 group, *Orpinomyces* genus and *Neocallimastix* genus; (4) M30 and M50 groups, *Orpinomyces* genus and an unclassified taxon; and (5) M40 group, *Orpinomyces* genus, *Neocallimastix* genus, and *Piromyces* genus. The addition of cotton straw had no effect on the dominance of *Orpinomyces* genus, but the relative abundance of *Caecomyces* genus and *Piromyces* genus decreased, and the relative abundance of the *Alternaria* genus increased. The *Alternaria* genus, which belongs to the phylum Ascomycota, degrades host cell walls through mechanical penetration and secretion of degradative enzymes, producing mycotoxins that act on the plasma membrane, mitochondria, chloroplasts and some metabolic enzymes (Kang et al., 2013). Some species are pathogenic to humans and animals in general, causing a variety of diseases (Sun, 2006). The genera *Cladosporium*, *Mycosphaerella*, and *Gibberella* are all plant pathogens. In this study, the relative abundance of *Gibberella* in the M30 group was higher than that of the CK1 group, Although *Gibberella* is not one of the dominant bacteria, its impact on rumen fermentation and health needs further study.

## 5 Conclusion

Increasing amounts of cotton straw in sheep rations had different effects on the structure of the bacterial and fungal communities in the rumen. The optimal inclusion level of cotton straw in the diet of ewes is 30%. With more than 40% cotton straw, the abundance of Proteobacteria in the rumen significantly increased, which increased the risk of adverse effects on the rumen microbiota. The addition of cotton straw increased the relative abundance of fungi in the phyla Ascomycota and Basidiomycota, which promoted the degradation of ligno-cellulose.

## Data Availability

All data has been made available at NCBI with accession number: PRJNA1287384.
